# Inpatient Outcomes Following a Return Visit to the Emergency Department: A Nationwide Cohort Study

**DOI:** 10.5811/westjem.2021.6.52212

**Published:** 2021-08-30

**Authors:** Chu-Lin Tsai, Dean-An Ling, Tsung-Chien Lu, Jasper Chia-Cheng Lin, Chien-Hua Huang, Cheng-Chung Fang

**Affiliations:** * National Taiwan University Hospital, Department of Emergency Medicine, Taipei, Taiwan; † National Taiwan University Hospital, College of Medicine, Department of Emergency Medicine, Taipei, Taiwan

## Abstract

**Introduction:**

Emergency department (ED) revisits are traditionally used to measure potential lapses in emergency care. However, recent studies on in-hospital outcomes following ED revisits have begun to challenge this notion. We aimed to examine inpatient outcomes and resource use among patients who were hospitalized following a return visit to the ED using a national database.

**Methods:**

This was a retrospective cohort study using the National Health Insurance Research Database in Taiwan. One-third of ED visits from 2012–2013 were randomly selected and their subsequent hospitalizations included. We analyzed the inpatient outcomes (mortality and intensive care unit [ICU] admission) and resource use (length of stay [LOS] and costs). Comparisons were made between patients who were hospitalized after a return visit to the ED and those who were hospitalized during the index ED visit.

**Results:**

Of the 3,019,416 index ED visits, 477,326 patients (16%) were directly admitted to the hospital. Among the 2,504,972 patients who were discharged during the index ED visit, 229,059 (9.1%) returned to the ED within three days. Of them, 37,118 (16%) were hospitalized. In multivariable analyses, the inpatient mortality rates and hospital LOS were similar between the two groups. Compared with the direct-admission group, the return-admission group had a lower ICU admission rate (adjusted odds ratio, 0.78; 95% confidence interval [CI], 0.72–0.84), and lower costs (adjusted difference, −5,198 New Taiwan dollars, 95% CI, −6,224 to −4,172).

**Conclusion:**

Patients who were hospitalized after a return visit to the ED had a lower ICU admission rate and lower costs, compared to those who were directly admitted. Our findings suggest that ED revisits do not necessarily translate to poor initial care and that subsequent inpatient outcomes should also be considered for better assessment.

## INTRODUCTION

Return emergency department (ED) visits pose a significant burden on both patients and healthcare providers, with approximately 5–10% of the patients returning to the ED within three days.^1^–^4^ Return ED visits are not only burdensome but costly, as one study found that the total cost of return ED visits was even higher than the total cost of all initial visits.^1^ Due to its clinical and economic ramifications, the rate of ED revisit has been used to measure potential lapses in initial emergency care.^5^ Recent studies, however, have begun to challenge this conventional wisdom. While the ED revisit rate is easy to measure, many factors may come into play, including factors related to the patient, the illness, the system, and finally to the clinician.^6^

It is estimated that only 5–10% of return ED visits are associated with potential deficiencies in care.^7^–^10^ More recent studies have examined patient outcomes after return ED visits as an alternative quality metric, such as hospitalization rates after ED revisits^11^–^16^ or even inpatient outcomes during the hospitalization after an ED revisit.^17^,^18^ Hospitalization rates after an ED revisit may also be problematic because ED admission rates per se are highly variable across EDs.^19^ Moreover, if the subsequent hospitalization after an ED revisit did not result in worse inpatient clinical outcomes due to a delay in admission, the assumption of poor care at the initial ED visit may be questionable.

Few studies to date (one of which focused on adults) have investigated inpatient outcomes among patients hospitalized during a return ED visit.^17^,^18^,^20^ The study with an adult cohort used data from two large, US states and found that patients who were admitted during an ED revisit had lower in-hospital mortality and intensive care unit (ICU) admission rates, compared with those who were admitted during the initial ED visit.^17^ To date, no studies have used nationwide data to address this issue. In the current study, we used nationwide data from a universal healthcare system to examine this topic. We investigated the patient characteristics, inpatient clinical outcomes, and resource use among patients who were admitted following a return visit to the ED, compared to those who were directly admitted during the index ED visit. We hypothesized that patients who were admitted after a revisit to the ED would experience similar inpatient outcomes and use similar inpatient resources.

## METHODS

### Study Design and Setting

We conducted a retrospective cohort study using data from the National Health Insurance Research Database (NHIRD) in Taiwan. The NHIRD contains all medical claims records from all clinical care settings covered by the National Health Insurance (NHI) program. The NHI is a mandatory, single-payer, government-run health insurance program that provides comprehensive health insurance to more than 99% of the 23 million Taiwanese residents.^21^ The NHIRD, maintained by the Ministry of Health and Welfare, has recorded comprehensive claims data in the NHI since 2000, including patient demographics, diagnoses, examinations, procedures, medications, and costs.^22^ The NHIRD is de-identified but contains a unique, encrypted personal identifier that allows researchers to link claims between outpatient, ED, and inpatient databases. We received a waiver for this analysis from our institutional review board.

### Study Population

We retrieved data from the registry of beneficiaries for the time period January 1, 2012–December 31, 2013. The sample for the current analysis contained approximately one-third of ED records, which were randomly extracted from the NHIRD via simple random sampling during the study period, including records of patients for their subsequent hospitalizations. This was the maximum amount of the data that could be requested. We excluded ED visits made by patients younger than 18 years, visits to urgent care clinics, ED transfers, or visits with unclear or missing time information.

**Population Health Research Capsule**

What do we already know about this issue?

*Emergency department (ED) revisits are used to measure potential lapses in emergency care. However, in-hospital outcomes are seldom examined after an ED revisit*.

What was the research question?

*We aimed to examine inpatient outcomes and resource use among patients hospitalized following a return visit to the ED*.

What was the major finding of the study?

*Patients hospitalized after an ED revisit had a lower ICU admission rate and incurred lower costs, compared to those directly admitted after the index ED visit*.

How does this improve population health?

*Revisits to the ED do not necessarily translate to poor initial care. Subsequent inpatient outcomes should also be considered for better assessment*.

We defined an index ED visit as an ED visit without a prior visit or hospitalization during the preceding three days. A return visit was defined as an ED revisit within 72 hours after discharge from the index ED. For multiple revisits within 72 hours, we selected only the first revisit. The unit of analysis was the visit, and one patient could have had multiple index visits during the study period. We chose to investigate early rather than late revisits because early revisits/readmissions have been shown to be more preventable and amenable to hospital-based interventions.^23^ We divided the cohort into two groups for comparison depending on the timing of hospitalization: (1) direct admissions, ie, patients who were admitted to the hospital during the index visit; and (2) return admissions, ie, those who were discharged from the ED at the index visit and were later hospitalized during the return visit to the ED.

### Variables

The NHIRD contains information on patient demographics, visit date and time, triage level, diagnostic codes (*International Classification of Diseases, Ninth Revision, Clinical Modification* [ICD-9-CM]), procedures, medications, ED disposition, hospital length of stay (LOS), and hospital disposition. We grouped the primary diagnosis field of ED and inpatient discharges into clinically meaningful categories using the ICD’s Clinical Classification Software.^24^ Comorbidities were also derived based on the ICD-9 codes using the Elixhauser Comorbidity index. This risk-adjustment tool has been validated extensively.^25^

In Taiwan, hospitals are classified into three distinct levels of accreditation according to the Joint Commission of Taiwan, including academic medical centers, regional hospitals, and community hospitals. The Taiwan Triage and Acuity Scales system is a computerized, five-level system with acuity levels 1 to 5 indicating resuscitation, emergent, urgent, less urgent, and non-urgent, respectively.^26^ The “untriaged” situation occurred in some of the psychiatric visits to community hospitals. The time of ED visit was classified as daytime (8 am – 4 pm), evening (4 pm – midnight), and night-time (12 am – 8 am).

### Outcome Measures

The outcome measures were inpatient mortality, intensive care unit (ICU) admission, LOS, and total inpatient costs in NT$ (New Taiwan dollar). We also examined the most common hospital discharge diagnoses among the two admission groups.

### Statistical Analysis

Summary statistics are presented as proportions (with 95% confidence intervals [CI]), means (with standard deviations), or medians (with interquartile ranges). We examined bivariate associations using Student’s t-test, Mann-Whitney tests, and chi-square tests, as appropriate. The inpatient outcomes (mortality and ICU admission) and resource use (LOS and cost) were analyzed by comparing the direct-admission group with the return-admission group. We used multivariable logistic and linear regression models to adjust for differences in patient mix. Although LOS and cost data were skewed, we did not transform the data because parametric methods are robust to non-normality with large samples.^27^ Instead, the associated multivariable linear-regression models were bootstrapped 1000 times to obtain the bias-corrected CIs.^28^ Potential confounding factors included age, gender, and Elixhauser comorbidities. All odds ratios (OR) and beta-coefficients are presented with 95% CIs. We performed all analyses using Stata 16.0 software (StataCorp, College Station, TX). All *P* values are two-sided, with *P* <0.05 considered statistically significant.

## RESULTS

After applying the exclusion criteria, there were 3,019,416 index ED visits during the two-year study period (Figure 1). Of them, 477,326 patients (16%) were admitted to the hospital following the index ED visit. Among the 2,504,972 ED discharges, 229,059 returned to the ED within three days. Of them, 37,118 (16%) were admitted to the hospital.

Table 1 shows the characteristics of the two hospitalization groups stratified by ED revisit status. Compared with the direct-admission group, patients in the return-admission group were slightly younger, predominantly male, and more likely to be triaged at a lower level (ie, less urgent). When revisiting the ED, the patients in the return-admission group were more likely to “move up” to regional hospitals or academic medical centers and were slightly more likely to show up at night, compared with the direct-admission group. In terms of revisit characteristics, most revisits occurred on day 1 after discharge, with a median time to revisit of 23 hours. Within the return-admission group, the triage levels went up upon revisit, compared with those at the index visits. However, the triage levels upon revisit in the return-admission group still appeared to be lower than those in the direct-admission group. Concerning comorbidities, in general, the return-admission group had fewer comorbid conditions, such as diabetes, hypertension, and congestive heart failure, compared with the direct-admission group. Of note, slightly more alcohol abuse and depression were present in the return-admission group.

Table 2 lists the hospital discharge diagnosis by ED visit status. The most common discharge diagnoses were quite similar between the two groups. Table 3 shows the study outcomes by ED revisit status. Compared with the direct-admission group, the return-admission group had lower inpatient mortality, a lower ICU admission rate, a shorter LOS, and incurred lower costs. Table 4 shows the study outcomes by ED visit status, after adjusting for age, gender, and 29 comorbidities. The differences in inpatient mortality and length of hospital stay became statistically non-significant between the two groups, while the return-admission group still had a lower ICU admission rate (adjusted OR, 0.78; 95% CI, 0.72–0.84), and incurred lower costs (adjusted difference, −5,198 NT$, 95% CI, −6,224 to −4,172).

## DISCUSSION

In this national ED and inpatient sample of 3,019,416 visits in Taiwan, we found that patients who were hospitalized after a return visit to the ED had a lower ICU admission rate and incurred lower costs, compared to those who were directly admitted during the index ED visit. Our data suggest that ED return admission does not necessarily reflect deficiencies in the initial ED care. Instead, because some clinical outcomes were better in the return-admission group than those in the direct-admission group, the clinicians at the initial ED encounter may have done what they were supposed to do, striking a balance between admitting sicker patients and safely discharging less-sick patients.

Our findings are consistent with previous studies that reported a less-ill revisit cohort compared with those without a prior ED visit.^17^,^29^ Both studies indicated that patients who returned to the ED were more likely to be uninsured, had fewer comorbidities, lower triage acuity, and similar or lower hospital admission rates.^17^,^29^ Our study extends these findings to a non-US population with universal health insurance coverage, suggesting these findings were not likely to be explained by lack of insurance alone. Given universal coverage, patients may choose to return to the ED for a quick assessment instead of scheduled outpatient follow-up. Of note, it is estimated that one-third of the revisits occurred at a different ED.^1^,^4^ Our study included both same- and different-hospital revisits in the entire nation, which may increase the likelihood of capturing more revisits and frequent ED users who may prefer the ED as a site of care.^30^,^31^ Despite the suggestion that some revisit patients appeared less ill, they might still prefer hospitalization as demonstrated by the similar hospitalization rates between the two groups. Again, this may reflect a shared decision-making process between patients and providers, which adds to the variation of revisit admission rates, undermining its validity as a quality metric.^19^

As EDs worldwide are seeing more and sicker patients, emergency physicians must make an appropriate decision to admit patients who are most likely to benefit from inpatient resources. After prioritizing patients, some will be sent home with certain risks of treatment failure, for example, prescribing antibiotics for pneumonia with outpatient follow-up. As shown in our data, although revisit patients had a higher acuity level compared with their prior visits,^32^ the revisit acuity was still lower than those who were admitted in the first place, suggesting a small and reasonable fraction of outpatient treatment failure. Furthermore, the lower ICU admission rates among the revisits did not suggest a harmful effect resulting from the decision to discharge at the index ED visits.

Consistent with a previous US study,^17^ we also found lower rates of ICU admission and costs among patients who returned to the ED, compared to those without a prior visit. Some of the mortality and LOS benefit among the revisit population was explained away by adjusting for age and comorbidities. Nonetheless, considering the additional evidence from ED revisits studies of inpatient outcomes, the ED revisit rate should not be used as a marker for ED quality.^5^ At a minimum, the subsequent inpatient outcome should be examined before adjudicating the initial ED quality of care. The slightly better inpatient outcomes among the revisit population also coincided with the finding of declined post-ED mortality among Medicare beneficiaries in the US who had visited an ED from 2009 to 2016.^33^ Taken together, these findings suggest that overall the post-ED outcomes of patients vising the ED have improved and that the rate of revisit as a quality metric must be evaluated from a patient outcome perspective.

## LIMITATIONS

This study has some potential limitations. First, we included only a limited number of patient outcomes in our analysis. There were additional clinical outcomes worth investigating that require more granular data, such as patient safety events and patient-reported outcomes. Second, we could not ascertain deaths after ED discharge. However, given the small number of post-ED deaths (0.12%) estimated from a prior study,^34^ the results should not have materially changed. Third, the data were somewhat aged and contained approximately one-third of the ED visits instead of the entire ED visit universe. However, this was the maximum amount of data that could be requested. As there have been no major policy changes regarding ED revisit in the past few years in Taiwan, the age of the data should have little, if any, influence on our results. Fourth, because we included only adult ED visits our results may not be generalizable to children. Fifth, caution should be exercised when applying the results to other healthcare settings. Finally, while we have adjusted for age, gender, and comorbidities when assessing inpatient outcomes, potential unmeasured confounders may still exist.

## CONCLUSION

In this national ED and inpatient database, patients who were hospitalized after a return visit to the ED within three days did not experience worse outcomes or use more resources than those who were directly admitted during the index ED visit. Our findings suggest that ED revisits per se do not necessarily translate to poor initial ED care and that inpatient outcomes should also be considered for better assessment. Further studies are needed to devise a feasible, sensitive, and specific quality-measure or screening algorithm (eg, return ICU admissions or return in-hospital mortality) for quality issues surrounding ED revisit.

## Figures and Tables

**Figure:**
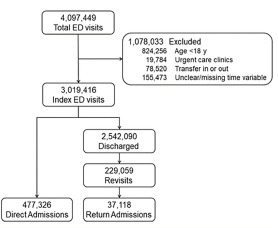
Flow diagram of the patient selection process. *ED*, emergency department; *y*, years old; *ED*, emergency department.

**Table 1:** Characteristics of hospitalizations stratified by revisit status.

Variable	Direct admission (n = 477,326)	Return admission (n = 37,118)	P-value
Age, mean (SD), yr	64.1 (19.2)	60.5 (19.8)	<0.0001
Age group, n (%)			<0.0001
18–64	220,490 (46.2)	19,904 (53.6)	
65+	256,836 (53.8)	17,214 (46.4)	
Female gender, n (%)	214,858 (45.0)	16,451 (44.3)	0.0098
Triage level at the index visit, n (%)			<0.0001
Level 1	27,579 (6.3)	774 (2.2)	
Level 2	90,108 (20.5)	5,202 (14.3)	
Level 3	258,554 (58.8)	23,813 (65.5)	
Level 4	44,608 (10.1)	4,847 (13.3)	
Level 5	2,869 (0.6)	264 (0.7)	
Untriaged (some psychiatric visits)	16,154 (3.7)	1,452 (4.0)	
Level of hospital accreditation, n (%)			<0.0001
Academic medical center	146,959 (30.8)	11,606 (31.3)	
Regional hospital	250,217 (52.4)	20,846 (56.2)	
Community hospital	80,150 (16.8)	4,666 (12.5)	
Weekend or holiday, n (%)	142,882 (29.9)	11,004 (29.7)	0.2434
Time of ED visit, n (%)			0.0195
Daytime (8 am – 4 pm)	215,492 (45.2)	16,713 (45.0)	
Evening (4 pm – 12 am)	184,756 (38.7)	14,211 (38.3)	
Night-time (12 am – 8 am)	77,078 (16.1)	6,194 (16.7)	
Day of revisit, n (%)			
Day 1	NA	19,499 (52.5)	
Day 2	NA	11,205 (30.2)	
Day 3	NA	6,414 (17.3)	
Time to ED revisit, median (IQR), hours	NA	23 (12–43)	
Revisit triage level, n (%)			
Level 1	NA	1,618 (4.9)	
Level 2	NA	6,269 (18.8)	
Level 3	NA	21,680 (64.9)	
Level 4	NA	3,013 (9.0)	
Level 5	NA	129 (0.4)	
Untriaged (some psychiatric visits)	NA	686 (2.0)	
Two or more comorbidities, n (%)	191,821 (40.2)	13,732 (37.0)	<0.001
Selected comorbidity, n (%)			
Congestive heart failure	33,599 (7.0)	2,192 (5.9)	<0.0001
Hypertension	96,283 (20.2)	7,240 (19.5)	0.0021
Chronic pulmonary disease	50,369 (10.5)	3,873 (10.4)	0.4757
Diabetes, uncomplicated	94,169 (19.7)	7,036 (19.0)	0.0003
Diabetes, complicated	19,235 (4.0)	1,398 (3.8)	0.0127
Liver disease	26,181 (5.5)	2,272 (6.1)	<0.0001
Metastatic cancer	25,224 (5.3)	1,732 (4.7)	<0.0001
Solid tumor without metastasis	61,675 (12.9)	4,045 (10.9)	<0.0001
Fluid and electrolyte disorders	41,834 (8.8)	3,268 (8.8)	0.7924
Alcohol abuse	3,817 (0.8)	403 (1.1)	<0.0001
Depression	2,255 (0.5)	219 (0.6)	0.0016

*IQR*, interquartile range; *ED*, emergency department; *SD*, standard deviation.

**Table 2:** Most common hospitalization diagnoses by revisit status.

Discharge diagnosis	Direct admission (n = 477,326) n (%)	Return admission (n = 37,118) n (%)
Pneumonia	43,782 (9.2)	2,984 (8.0)
Urinary tract infection	31,149 (6.5)	2,672 (7.2)
Sepsis	23,184 (4.9)	1,903 (5.1)
Acute cerebrovascular disease	19,588 (4.1)	1,406 (3.8)
Gastrointestinal hemorrhage	13,763 (2.9)	
Biliary tract disease		1,363 (3.7)

**Table 3:** Study outcomes by revisit status (unadjusted).

Variable	Direct admission (n = 477,326)	Return admission (n = 37,118)	P value
In-hospital mortality, n (%)	20,003 (4.2)	1,447 (3.9)	0.0067
ICU admission, n (%)	13,056 (2.7)	793 (2.1)	<0.0001
Length of hospital stay, days			
Mean (SD)	9.4 (8.2)	9.1 (8.0)	<0.0001
Median (IQR)	7 (4–11)	7 (4–11)	<0.0001
Total cost, NT$			
Mean (SD)	55,758 (99,425)	47,954 (89,644)	<0.0001
Median (IQR)	26,770 (14,272–56,786)	22,013 (11,468–46,875)	<0.0001

*IQR*, interquartile range; *SD*, standard deviation; *ICU*, intensive care unit, *NT$*, New Taiwan dollar.

**Table 4:** Study outcomes by revisit status, adjusted.

Outcome measures, point estimate (95% CI)*	Direct admission	Return admission
In-hospital mortality, OR	Reference	1.06 (0.99–1.12)
ICU admission, OR	Reference	0.78 (0.72–0.84)
Length of hospital stay, days	Reference	0.03 (−0.05 to 0.12)
Total cost, NT$	Reference	−5,198 (−6,224 to −4,172)

* Adjusted for age, gender, and 29 Elixhauser comorbidities.

*CI*, confidence interval; *OR*, odds ratio; *ICU*, intensive care unit; *NT$*, New Taiwan dollar.
